# Molecular identification of resistance to organophosphates and carbamates in *Aedes aegypti* of different physiological ages in a cemetery in Peru

**DOI:** 10.17843/rpmesp.2025.423.14471

**Published:** 2025-09-29

**Authors:** Rosa Elena Santillan-Valdivia, Mariano Manuel Yañez Cesti, Ruth Noemi Neyra-Palacios, Archi Alejandro Ruiz-Polo

**Affiliations:** 1 Centro de Investigación y Capacitación en Entomología, Dirección Sub Regional de Salud Luciano Castillo Colonna, Sullana, Piura, Peru. Centro de Investigación y Capacitación en Entomología Dirección Sub Regional de Salud Luciano Castillo Colonna Sullana Piura Peru; 2 Dirección Sub Regional de Salud Luciano Castillo Colonna, Sullana, Piura, Peru. Dirección Sub Regional de Salud Luciano Castillo Colonna Sullana Piura Peru

**Keywords:** Aedes aegypti, Parity, Insecticide resistance, Organophosphates, Carbamates, Acetylcholinesterase, PCR

## Abstract

In 2024, a cross-sectional, quantitative, and descriptive study was conducted to molecularly identify resistance to organophosphates and carbamates in *Aedes aegypti* of different physiological ages in a cemetery in northern Peru. Female specimens were captured, taxonomically identified, their ovaries dissected, and the parity percentage calculated. Likewise, the legs of nulliparous, parous, and gravid females were dissected, DNA was extracted, and PCR was performed to detect the mutant ACEr gene associated with resistance to organophosphates and carbamates. A total of 218 (100%) females were captured, with a nulliparity index of 37,61% and a parity index of 62,39%, of which 13,23% of parous females were gravid at the time of capture. The ACEr gene was detected in all three physiological age groups. It is inferred that in the analyzed cemetery, there are *Aedes aegypti* populations carrying the genetic mutation conferring resistance to organophosphate and carbamate insecticides.

## INTRODUCTION

Vector-borne diseases are responsible for more than one million deaths annually worldwide ^1^, with *Aedes aegypti* being one of the mosquitoes of most significant medical importance due to its ability to transmit viruses responsible for diseases such as dengue, chikungunya, Zika, and yellow fever ^2^. In addition, it is a potential vector for the Venezuelan equine encephalitis virus, the Mayaro virus, and the West Nile virus ^3^.

In recent decades, the incidence of dengue has increased significantly, with a 30% increase in its frequency ^4^. This pattern was also observed in the department of Piura ^5^, which is relevant considering the emergence of the cosmopolitan genotype of the DENV-2 serotype, first identified during an outbreak in 2019 ^6^.

In 2019, it was reported that various Peruvian populations of *Aedes aegypti* showed resistance to dichloro-diphenyl-trichloro-ethane (DDT) and pyrethroid insecticides, and in most cases, to organophosphates. Nevertheless, some populations in the department of Piura remained susceptible to malathion ^7^. In 2024, a report from the National Institute of Health (INS) on the resistance of *Aedes aegypti* to insecticides, including malathion, showed that mosquito populations had developed resistance to this compound, suggesting the need for its rotation. Despite this, the same report mentions that, after field tests, this insecticide is still effective for the control of the dengue vector ^8^. Faced with this uncertainty, a key tool for identifying resistant mosquito populations is the detection of mutations in their genetic material ^9^, as these can be related to ecological and physiological aspects that influence their ability to tolerate chemical compounds ^10^^,^^11^.

In this context, the present study contributes to the anticipation and mitigation of entomo-epidemiological risk in urban areas of Peru, since cemeteries constitute persistent and high-population-density breeding sites for *Aedes aegypti*. Likewise, the detection of the mutation associated with resistance to organophosphate and carbamate insecticides in females of different physiological ages allows for understanding the mechanisms of genetic transfer between generations. This is fundamental information for vector control strategies, as the presence of populations with insecticide resistance mutations reduces the effectiveness of vector control. The objective of this research is to perform the molecular identification of resistance to organophosphates and carbamates in *Aedes aegypti* of different physiological ages in a cemetery in Peru during 2024.

**KEY MESSAGES**

Motivation for the study: In northern Peru, studies on the molecular detection of insecticide resistance in *Aedes aegypti* at different physiological ages are scarce.

Main findings: Between July and August 2024, in a cemetery in northern Peru, juvenile and long-lived populations of *Aedes aegypti* were identified that presented genetic resistance to organophosphate and carbamate insecticides.

Public health implications: The findings show that in a cemetery in the department of Piura, there are populations of *Aedes aegypti* with genetic resistance to organophosphate and carbamate insecticides, for which Health Directorates need to rethink their vector control strategies.

## THE STUDY

### Study design and area

A non-experimental study with a quantitative approach, descriptive level, and cross-sectional type was conducted between July and August 2024, in the San José Cemetery of Sullana (Latitude -4.88674° / Longitude -80.68139°), located in northern Peru, in the department of Piura (supplementary material).

### Entomological captures and taxonomic identification

From 9:00 a.m. to 1:00 p.m., adult culicid mosquitoes were captured using the resting capture method ^12^. The captured specimens were placed in entomological cups and transported to the bioassay area of the Entomology Research and Training Center - CICE. Afterward, they were exposed to ethyl acetate-impregnated cotton for 8 minutes. Then, they were transferred to Petri dishes and identified to the species level using taxonomic keys ^13^.

### Ovary dissection

The taxonomically identified specimens were placed on sterile slides, where the ovaries were dissected with the assistance of a stereomicroscope ^14^.

### Microscopy and stereomicroscopy of ovaries

An analysis was performed using microscopy and stereomicroscopy to classify nulliparous and parous females, observing coiled and uncoiled tracheoles, respectively. The dissections were performed with entomological forceps, No. 0.00 stylets, and 0.65% saline solution, using a transmitted light binocular microscope (model Axiostar 1122-100) and a binocular stereomicroscope (model Stemi DV4), both from the ZEISS brand and coupled to the camera of an Honor X7 smartphone (model CMA-LX3). The number of nulliparous and parous females was determined by the nulliparity and parity index, considering gravid females as parous, according to the formula:

*Parity/Nulliparity Index(%)= A/B ×100*

Where: 

A = number of parous and/or nulliparous females 

B = total number of females captured

### Leg dissection and DNA extraction

Six groups of females were formed, each consisting of 10 specimens distributed into two groups of gravid, two groups of parous, and two groups of nulliparous females. Then, the legs were dissected from the coxa to the claws and washed with ultrapure water (UPW). 300 µL of 0.9% sodium chloride (NaCl) and 600 µL of Lysis Buffer from the Zymo Biomics DNA Miniprep Kit (Cat. D4300) were added; they were crushed with sterile grinders and the tubes were incubated at 56 ºC for 25 minutes. Afterward, the manufacturer’s instructions were followed, modifying the cell lysis step by omitting the ZR BashingBead™ Lysis Tubes.

### PCR for the detection of the ACEr gene

The amplification of the ACEr gene, with a size of 190 bp, was performed using the PCR GoTaq™ G2 Flexi DNA Polymerase kit (Promega M7801), following the manufacturer’s instructions. The forward and reverse primers described by Loaiza ^9^ were used. The final reaction volume was 25 µL: 11.25 µL of nuclease-free water, 5 µL of 1X buffer, 1.5 µL of MgCl2 (1.5 mM), 0.5 µL of dNTPs (200 µM), 1.25 µL of ACER Forward primer (10 µM), 1.25 µL of ACER Reverse primer (10 µM), 0.25 µL of GoTaq Polymerase enzyme (1 U/reaction), and 4 µL of total DNA. The thermal and amplification cycle conditions were: initial denaturation at 94 °C for 5 minutes, followed by 30 cycles with the following steps: denaturation at 94 °C for 30 seconds, hybridization at 60 °C for 30 seconds, and extension at 72 °C for 1 minute and 30 seconds. At the end of the cycle, a post-extension was performed at 72 °C for 5 minutes, and the mixture was kept for up to 24 hours at 4 °C. A PCR product with a size of 190 base pairs (bp) was expected.

### Agarose gel electrophoresis

The obtained PCR products (amplicons) were visualized by electrophoresis on a 2% agarose gel in 1X TAE solution (Tris-Acetate-EDTA), stained using 4.5 µL of Ethidium Bromide and 10 µL of the extracted DNA sample. For DNA migration, 4 µL of 6X loading buffer and 2 µL of a 1000 bp (1kb) molecular weight marker (Opti-DNA Marker G016) were used. This procedure confirmed the amplification of the ACEr gene sequence.

### Ethical considerations

The study was conducted in compliance with current regulations and with the corresponding institutional authorization, ensuring respect for ethical principles throughout the research process.

## FINDINGS

### Physiological age

**Figure 1:**
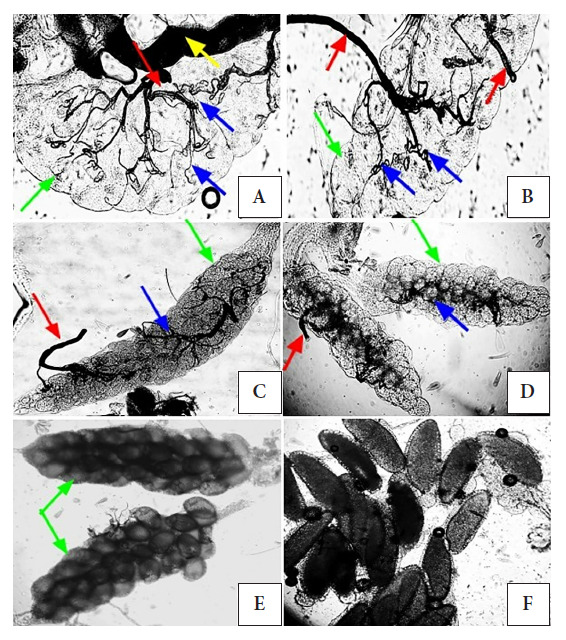
Gonotrophic evaluation with a 10X / 0.25 objective in Aedes aegypti. A. Left ovary of a parous female in an inverted position (green arrow indicates the ovary with oocytes, red arrow indicates tracheae, blue arrow indicates uncoiled tracheoles, and yellow arrow indicates a fragment of the Malpighian tubule). B. Left ovary of a nulliparous female in an inverted position (green arrow indicates the ovary with oocytes, red arrow indicates tracheae, and blue arrow indicates coiled tracheoles). C. Left ovary of a parous female in the position opposite to inverted (green arrow indicates the ovary with oocytes, red arrow indicates tracheae, and blue arrow indicates uncoiled tracheoles). D. Left ovary of a nulliparous female in the position opposite to inverted (green arrow indicates the ovary with oocytes, red arrow indicates tracheae, and blue arrow indicates coiled tracheoles). E. Ovary of a subgravid female (green arrow indicates ovaries with fertilized oocytes). F. Developed eggs in a gravid female. In A, B, C, and D, the unfertilized oocytes are differentiated from the fertilized ones by their translucent color and size.

Figure 1 shows the ovaries and their morphological structures (tracheae and tracheoles), obtained from female *Aedes aegypti* mosquitoes captured in the San José cemetery of Sullana between July 27 and August 20. The analysis of ovaries revealed that out of 218 females (100%), 82 (37.6%) were found to be nulliparous and 136 (62.4%) were parous. However, 13.2% (n=18) of the parous females were gravid at the time of their capture. The results obtained suggest that *Aedes aegypti* was in an active reproductive phase in the cemetery, as more than 50% of the females exhibited a parous state without eggs and with eggs.

### Molecular identification of resistance to organophosphates and carbamates

**Figure 2:**
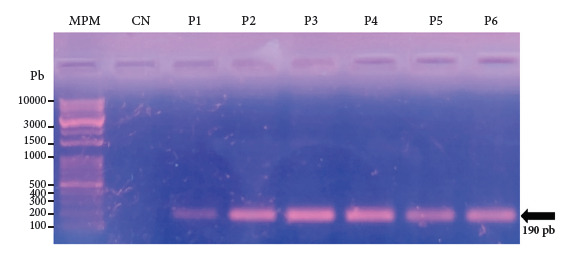
2% agarose gel electrophoresis of the PCR products targeting the ACEr gene obtained from adult female specimens of Aedes aegypti from the San José de Sullana cemetery in northern Peru. P1: Pool of 8 gravid females. P2: Pool of 10 gravid females. P3 and P4: Pool of 10 parous females. P5 and P6: Pool of 10 nulliparous females

Figure 2 shows the amplification of the ACEr gene in 100% (n=58) of the female mosquitoes selected for molecular analysis, indicating resistance to organophosphate and carbamate insecticides.

## DISCUSSION

In the present study, it was observed that more than 50% of the analyzed females had uncoiled tracheoles, and some were found to be gravid. Females classified as nulliparous (juvenile) are those without a blood meal with coiled tracheoles, while parous females (long-lived) are those that have completed one oviposition and have uncoiled tracheoles ^14^. The physiological age of culicid mosquitoes can be estimated by analyzing the tracheolar structure of their ovaries ^15^. According to Cabezas ^10^, a female that feeds completely on blood will lay up to 200 eggs, in an interval of two to three days. Badil et al. ^16^ point out that in *Aedes aegypti*, the interval between blood intake and oviposition can be as short as three days, under optimal temperature conditions and host availability. Morin et al. ^17^^)^ highlight that blood intake in mosquitoes directly influences the duration of gonotrophic cycles, so abdominal distension during feeding is a key factor. However, previous studies indicate that blood digestion is influenced by temperature and humidity, such that at low temperatures the gonotrophic cycle is lengthened, while at high temperatures it is shortened ^18^. Salas ^19^^)^ describes that the gonotrophic cycle of *Aedes aegypti* can last up to five days, although other studies suggest it can vary up to 20 days ^20^.

Regarding the detection of the ACEr gene, it was found in 20 nulliparous (juvenile) females, 20 parous (long-lived) females, and 18 gravid of the parous (long-lived) females. This finding is consistent with the results of Loaiza ^9^, who detected the same gene in *Aedes aegypti* populations in Mexico. A similar study showed that *Aedes aegypti* populations exhibit resistance to insecticides such as deltamethrin, permethrin, malathion, chlorpyrifos, temephos, and bendiocarb, attributed to high activity of detoxifying enzymes, such as cytochrome P450 and glutathione S-transferase, as well as a decrease in the sensitivity of the acetylcholinesterase enzyme ^(^^21^. According to Sánchez and Salceda ^22^, acetylcholinesterase (AChE) is a key enzyme for the functioning of the nervous system in insects and other organisms; its main function is to break down acetylcholine, an essential neurotransmitter for the transmission of nerve impulses at synapses. According to Pascual and López ^23^, in insects, AChE is located in neurons and motor endplates, where it regulates neuronal signaling.

In 2024, susceptibility to organophosphates and resistance mechanisms were evaluated in *Aedes aegypti* populations in Colombia, finding resistance to the insecticides temephos, malathion, pirimiphos-methyl, and fenitrothion induced by insensitivity caused by mutations in genes encoding the enzymes glutathione S-transferases, α-esterases, mixed-function oxidases, and acetylcholinesterase ^24^. In 2021, in Panama, populations of *Aedes aegypti* and *Aedes albopictus* were identified that exhibit resistance to insecticides of the pyrethroid, carbamate, and organophosphate classes ^25^. In Venezuela, alteration in the expression of enzymes involved in the functional activity of insecticides, such as glutathione-S-transferase and acetylcholinesterase, has been reported, which has led to the development of resistance to organophosphates in *Anopheles aquasalis*, the main vector of malaria in that country ^26^. A study conducted in Cuba in 1986 reported an increase in the densities of *Culex quinquefasciatus* in urban areas, which later led to the identification of resistance to malathion in two populations of this mosquito ^27^. Therefore, the results of this study, together with reports from other South American countries and the 2024 report from the National Institute of Health (INS) of Peru on the resistance of *Aedes aegypti* to malathion ^8^, reinforce and complement the findings previously described by the INS.

The strength of the study is based on the fact that by using molecular techniques in *Aedes aegypti* vector control strategies, it will be possible to perform genetic monitoring to detect the appearance of mutations in genes related to insecticide resistance, making it possible to anticipate the need to replace and/or rotate them with more effective insecticides.

The present investigation has an important limitation that must be acknowledged. The entomological captures were carried out in a cemetery located in an urban area, which restricts the possibility of generalizing that *Aedes aegypti* populations from cemeteries in rural areas also present genetic characteristics associated with resistance.

In conclusion, in northern Peru, specifically in the San José cemetery of Sullana in the department of Piura, the existence of *Aedes aegypti* populations, both juvenile adults (nulliparous) and long-lived (parous and gravid), has been identified, which possess a mutation in acetylcholinesterase, conferring them resistance to organophosphate and carbamate insecticides.

## References

[1] Padilla JC, Pardo R, Molina JA (2017). Manejo integrado de los riesgos ambientales y el control de vectores una nueva propuesta para la prevención sostenible y el control oportuno de las enfermedades transmitidas por vectores. Rev Biomed.

[2] Hill C, Kafatos FC, Stansfield SK, Collins FH (2005). Arthropod-borne diseases vector control in the genomics era. Nat Rev Microbiol.

[3] Vanlandingham DL, McGee CE, Klinger KA, Vessey N, Fredregillo C, Higgs S (2007). Short report Relative susceptibilties of South Texas mosquitoes to infection with West Nile virus. Am J Trop Med Hyg.

[4] Pin VEP, Tumbaco IJL, Hernández NKJ, Palma SGC (2019). Factores de riesgos que influyen en las enfermedades vectoriales factores de riesgo. Rev Sinap.

[5] Gobierno regional de Piura (2024). Alerta epidemiológica por epidemia de dengue en la región de Piura.

[6] García MP, Padilla C, Figueroa D, Manrique C, Cabezas C (2022). Emergence of the Cosmopolitan genotype of dengue virus serotype 2 (DENV2) in Madre de Dios, Perú, 2019. Rev Peru Med Exp Salud Publica.

[7] Pinto J, Palomino M, Mendoza-Uribe L, Sinti C, Liebman KA, Lenhart A (2019). Susceptibility to insecticides and resistance mechanisms in three populations of Aedes aegypti from Peru. Parasit Vectors.

[8] Instituto Nacional de Salud (2024). Resistencia de Aedes aegypti a los insecticidas, nuevas alternativas.

[9] Loaiza Becerra MH (2002). Detección del gen de resistencia acetilcolinesterasa (ACEr) a insecticidas organofosforados y carbamatos en Aedes aegypti (L.) en diez localidades del estado de Veracruz, México.

[10] Cabezas C (2005). Dengue in Peru: Support for its diagnosis and control. Rev Peru Med Exp Salud Publica.

[11] Howard O, Dyar HG, Knab F (1912). The Mosquitoes of North and Central America and the West Indies.

[12] Organización Mundial de la Salud (1975). Manual on practical entomology in Malaria. Part. II.

[13] Consoli R, Laureco T, Oliveira L (1994). Principales mosquitos de importancia sanitaria en Brasil.

[14] Detinova TS (1962). Age-grouping methods in Diptera of medical importance with special reference to some vectors of malaria. Monogr Ser World Health Organ.

[15] Luis Arismendiz LD (2015). Edad fisiológica de hembras de Lutzomyia spp. (Diptera: Psychosidae) en el distrito de Sapillica, provincia de Ayabaca, región Piura en el 2013.

[16] Badii MH, Landeros J, Cerna E, Abreu JL (2007). Ecología e Historia del Dengue en las Américas. Daena: International J Good Conscience.

[17] Morin C, Comrie A, Ernst K (2013). Climate and Dengue Transmission Evidence and Implications. Environ Health Perspect.

[18] Goindin D, Delannay C, Ramdini C, Gustave J, Fouque F (2015). Parity and longevity of Aedes aegypti according to temperatures in controlled conditions and consequences on dengue transmission risks. PLoS One.

[19] Salas M (1993). Ciclo gonotrófico, tasa de supervivencia y estructura de edades de Aedes aegypti, en la zona Metropolitana de Monterrey, Nuevo León, México.

[20] Conde A (2003). Estudio de la longevidad y el ciclo gonotrófico del Aedes (Stegomyia) aegypti (LINNAEUS, 1762), cepa Girardot (Cundinamarca) en condiciones de laboratorio.

[21] López-Solís AD, Castillo-Vera A, Cisneros J, Solís-Santoyo F, Penilla-Navarro RP, Black IV (2020). Resistencia a insecticidas en Aedes aegypti y Aedes albopictus (Diptera Culicidae) de Tapachula, Chiapas, México. Salud Publica Mex.

[22] Sánchez CG, Salceda R (2008). Enzimas polifuncionales: El caso de la acetilcolinesterasa. Revista de Educación Bioquímica.

[23] Pascual Villalobos MJ, López Belchí MD (2015). Toxicidad volátil de monoterpenoides y mecanismos bioquímicos en insectos plaga del arroz almacenado.

[24] García Leal YJ (2024). Susceptibilidad a organofosforados e identificación de mecanismos de resistencia en poblaciones de Aedes aegypti (Díptera: Culicidae) de los municipios de Ayapel y Planeta Rica (Córdoba, Colombia).

[25] Tuñon AL (2009). Determinación de la resistencia a insecticidas y sus mecanismos bioquímicos en poblaciones de Aedes aegypti y Aedes albopictus del Distrito De Panamá, República de Panamá.

[26] Molina D, Figueroa LE (2009). Resistencia metabólica a insecticidas organofosforados en Anopheles aquasalis Curry 1932, municipio Libertador, estado Sucre, Venezuela. Rev Biomed.

[27] Bisset JA, Rodríguez MM, Diaz D, Ortiz E, Marquetti MC, Hemingway J (1990). The mechanisms of organophosphate and carbamate resistance in Cx quinquefasciatus (Diptera: Culicidae) from Cuba. Bull Entomol Res.

