# Supplementary material for: Molecular identification of resistance to organophosphates and carbamates in Aedes aegypti of different physiological ages in a cemetery in Peru
Source: Rev Peru Med Exp Salud Publica. 2025 Sep 29;42(3):312–7. doi: 10.17843/rpmesp.2025.423.14471 (PMC12679977; doi:10.17843/rpmesp.2025.423.14471)
Supplement: Supplementary material. — Available in the electronic version of the RPMESP. [file rpmesp-42-03-14471-s001.docx]

**Material suplementario**


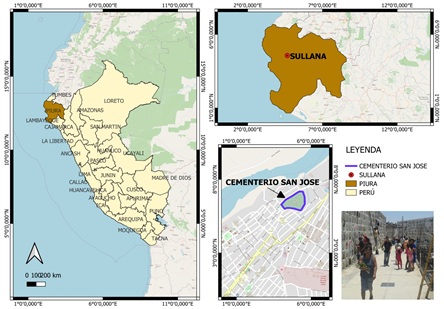


**Figura 1.** Ubicación geográfica del cementerio San José de Sullana donde se capturaron los especímenes de *Aedes aegypti* analizados.
