# Supplementary material for: Molecular identification of resistance to organophosphates and carbamates in Aedes aegypti of different physiological ages in a cemetery in Peru
Source: Rev Peru Med Exp Salud Publica. 2025 Sep 29;42(3):312–7. doi: 10.17843/rpmesp.2025.423.14471 (PMC12679977; doi:10.17843/rpmesp.2025.423.14471)
Supplement: Translation

# Translation: Identificación molecular de la resistencia a organofosforados y carbamatos en *Aedes aegypti* de distintas edades fisiológicas en un cementerio del Perú

**Authors:** Rosa Elena Santillan-Valdivia, Mariano Manuel Yañez Cesti, Ruth Noemi Neyra-Palacios, Archi Alejandro Ruiz-Polo

**Affiliations:** 1 Centro de Investigación y Capacitación en Entomología, Dirección Sub Regional de Salud Luciano Castillo Colonna, Sullana, Piura, Perú.; 2 Dirección Sub Regional de Salud Luciano Castillo Colonna, Sullana, Piura, Perú.

**Keywords:** Aedes aegypti, Paridad, Resistencia a insecticidas, Organofosforados, Carbamatos, Acetilcolinesterasa, PCR

## Abstract

En 2024 se realizó un estudio transversal, cuantitativo y descriptivo para identificar molecularmente la resistencia a organofosforados y carbamatos en *Aedes aegypti* de distintas edades fisiológicas en un cementerio del norte de Perú. Se capturaron mosquitos hembras, se identificaron taxonómicamente, se diseccionaron sus ovarios y se estimó el porcentaje de paridad. Asimismo, se diseccionaron las patas de hembras nulíparas, paridas y grávidas, de las cuales se extrajo ADN, y se realizó la PCR del gen mutante ACEr asociado con la resistencia a organofosforados y carbamatos. Se capturaron 218 (100%) hembras con un índice de nuliparidad del 37,6% y paridad del 62,4%, observándose que el 13,2% de las paridas se encontraban grávidas al momento de su captura. Además, se identificó la presencia del gen ACEr en las tres edades fisiológicas evaluadas. Estos hallazgos sugieren que en el cementerio analizado existen poblaciones de *Aedes aegypti* con la mutación genética asociada a la resistencia a insecticidas organofosforados y carbamatos.

## INTRODUCCIÓN

Las enfermedades transmitidas por vectores son responsables de más de un millón de muertes anuales en todo el mundo ^1^, siendo *Aedes aegypti* uno de los moquitos con mayor importancia médica debido a su capacidad de transmitir virus responsables de enfermedades como el dengue, chikungunya, zika y fiebre amarilla ^2^. Además, es un potencial vector del virus de la encefalitis equina venezolana, el virus Mayaro y el virus del Nilo Occidental ^3^.

En las últimas décadas, la incidencia del dengue ha aumentado significativamente, con un incremento del 30% en su frecuencia ^4^, un patrón observado en el departamento de Piura ^5^, lo cual es relevante considerando la aparición del genotipo cosmopolita del serotipo DENV-2, identificado por primera vez durante un brote en 2019 ^6^.

En 2019 se reportó que diversas poblaciones peruanas de *Aedes aegypti* presentaban resistencia a insecticidas dicloro difenil tricoloroetano (DDT), piretroides y, en la mayoría de los casos a organofosforados. No obstante, algunas poblaciones del departamento de Piura aún se mantenían susceptibles al malathion ^7^. Sin embargo, en 2024, un informe del Instituto Nacional de Salud (INS), sobre la resistencia de *Aedes aegypti* a insecticidas, incluido el malathion, evidenció que poblaciones del mosquito habian desarrollado resistencia a este compuesto, sugiriendo la necesidad de su rotación. A pesar de ello, el mismo informe menciona qué, tras pruebas de campo realizadas, este insecticida aún sigue siendo eficaz para el control del vector del dengue ^8^. Frente a esta incertidumbre, una herramienta clave para identificar poblaciones de mosquitos resistentes, es la detección de mutaciones en su material genético ^9^, ya que estas pueden estar relacionadas con aspectos ecológicos y fisiológicos que influyen en su capacidad para tolerar compuestos químicos ^10^^,^^11^.

En ese contexto, el presente estudio contribuye a la anticipación y mitigación del riesgo entomoepidemiológico en áreas urbanas del Perú, ya que los cementerios constituyen criaderos persistentes y de alta densidad poblacional de *Aedes aegypti*. Asimismo, la detección de la mutación asociada a la resistencia de insecticidas organofosforados y carbamatos en hembras de distintas edades fisiológicas permite comprender los mecanismos de transferencia genética entre generaciones. Lo cual es información fundamental para las estrategias de control vectorial, ya que la presencia de poblaciones con mutaciones de resistencia a insecticidas reduce la efectividad del control vectorial.

El objetivo de esta investigación es realizar la identificación molecular de la resistencia a organofosforados y carbamatos en *Aedes aegypti* de distintas edades fisiológicas en un cementerio de Perú durante el 2024.

**MENSAJE CLAVE**

Motivación para realizar el estudio: En el norte de Perú, los estudios sobre la detección molecular de resistencia a insecticidas en *Aedes aegypti* en distintas edades fisiológicas son escasos.

Principales hallazgos: Entre julio y agosto del 2024, en un cementerio del norte de Perú, se identificaron poblaciones juveniles y longevas de *Aedes aegypti* que presentaban resistencia genética a insecticidas organofosforados y carbamatos.

Implicancias para la salud pública: Los hallazgos muestran que en un cementerio del departamento de Piura existen poblaciones de *Aedes aegypti* con resistencia genética a insecticidas organofosforados y carbamatos, por los que las Direcciones de Salud necesitan replantear sus estrategias de control vectorial.

## EL ESTUDIO

### Diseño y área del estudio

Se realizó un estudio con diseño no experimental, enfoque cuantitativo, nivel descriptivo - tipo transversal, entre julio y agosto del año 2024; en el Cementerio San José de Sullana (Latitud -4.88674° / Longitud -80.68139°), ubicado al norte en Perú, en el departamento de Piura (material suplementario).

### Capturas entomológicas e identificación taxonómica

En un horario de 9:00 am a 1:00 pm, se capturaron mosquitos adultos culícidos mediante el método de captura en reposo ^12^. Los ejemplares capturados fueron colocados en vasos entomológicos y transportados al área de bioensayos del Centro de Investigación y Capacitación en Entomología - CICE. Después, se expusieron a acetato de etilo impregnado en algodón durante 8 minutos. Luego, se transfirieron a placas de Petri y se identificaron a nivel de especie mediante claves taxonómicas ^13^.

### Disección de ovarios

Los especímenes identificados taxonómicamente se colocaron sobre láminas portaobjetos estériles, donde se diseccionaron los ovarios con asistencia de un estereomicroscopio ^14^.

### Microscopía y estereomicroscopía de ovarios

Se realizó un análisis mediante microscopía y estereomicroscopía para clasificar hembras nulíparas y paridas, observando traqueolas enrolladas y desenrolladas, respectivamente. Las disecciones se efectuaron con pinzas entomológicas, estiletes N.º 0,00 y solución salina al 0,65%, utilizando un microscopio binocular de luz transmitida (modelo Axiostar 1122-100) y un estereomicroscopio binocular (modelo Stemi DV4), ambos de la marca ZEISS y acoplados a la cámara de un smartphone Honor X7 (modelo CMA-LX3). El número de hembras nulíparas y paridas se determinó mediante el índice de nuliparidad y paridad, considerando a las hembras grávidas como paridas, según la fórmula:

Índice de paridad y/o nuliparidad = A/B*100

Dónde:

A= número de hembras paridas y/o nulíparas

B= número total de hembras capturadas 

### Disección de patas y extracción del ADN

Se formaron seis grupos de hembras, cada uno constituido por 10 ejemplares distribuidos en dos grupos de grávidas, dos de paridas y dos de nulíparas. Luego, se diseccionaron las patas desde la coxa hasta las uñas y se lavaron con agua ultrapura (AUP). Se añadieron 300 µL de cloruro de sodio (NaCl) al 0,9 % y 600 µL de Buffer de Lisis del Kit Miniprep Zymo Biomics DNA (Cat. D4300); se trituraron con maceradores estériles y los tubos se incubaron a 56 ºC durante 25 minutos. Después, se siguieron las instrucciones del fabricante modificando el paso de lisis celular al omitir los tubos ZR Bashing Bead™.

### PCR dirigida a la detección del gen ACEr

Se realizó la amplificación del gen ACEr, con un tamaño de 190 pb, utilizando el kit PCR GoTaq™ G2 Flexi DNA Polymerase (Promega M7801), siguiendo las instrucciones del fabricante. Se emplearon los cebadores forward y reverse, descritos por Loaiza ^9^. El volumen final de la reacción fue de 25 µL: 11,25 µL de agua libre de nucleasas, 5 µL de buffer 1X, 1,5 µL de MgCl_2_ (1,5 mM), 0,5 µL de dNTPs (200 µM), 1,25 µL de cebador Forward ACER (10 µM), 1,25 µL de cebador Reverse ACER (10 µM), 0,25 µL de la enzima GoTaq Polimerasa (1 U/reacción) y 4 µL de ADN total. Las condiciones térmicas y el ciclo de amplificación fueron: desnaturalización inicial a 94 °C durante 5 minutos, seguida de 30 ciclos con los siguientes pasos: desnaturalización a 94 °C por 30 segundos, hibridación a 60 °C por 30 segundos, y extensión a 72 °C por 1 minuto y 30 segundos. Al final del ciclo, se realizó una posextensión a 72 °C por 5 minutos y se conservó la mezcla hasta 24 horas a 4 °C. Se esperó un producto PCR con un tamaño de 190 pares de bases (pb).

### Electroforesis en gel de agarosa

Los productos de PCR (amplicones) obtenidos fueron visualizados mediante electroforesis en gel de agarosa al 2% en solución TAE 1X (Tris-Acetato-EDTA), con tinción utilizando 4,5 µL de bromuro de Etidio y 10 µL de la muestra de ADN extraído. Para la migración del ADN, se emplearon 4 µL de buffer de carga 6X y 2 µL de marcador de peso molecular de 1000 pb (1kb) (Opti-DNA Marker G016). Este procedimiento permitió confirmar la amplificación de la secuencia del gen ACEr.

### Consideraciones éticas

El estudio se llevó a cabo en cumplimiento de las normativas vigentes y con la autorización institucional correspondiente, asegurando el respeto a los principios éticos durante todo el proceso de investigación.

## HALLAZGOS

### Edad fisiológica

La figura 1 muestra los ovarios y sus estructuras morfológicas (tráqueas y traqueolas), obtenidos de zancudos hembras de *Aedes aegypti* capturadas en el cementerio San José de Sullana entre el 27 de julio y 20 de agosto. El análisis de ovarios reveló qué de 218 hembras (100%), 82 (37,6%) se hallaron nulíparas y 136 (62,4%) paridas. No obstante, el 13,2% (n=18) de las hembras paridas se encontraban grávidas en el momento de su captura. Los resultados obtenidos sugieren que *Aedes aegypti* se encontraba en una fase reproductiva activa en el cementerio, ya que más del 50% de las hembras exhibieron un estado parido sin huevos y con huevos.

**Figura 1:** Evaluación gonadotrófica con objetivo 10X / 0,25 en *Aedes aegypti.* A. Ovario izquierdo de hembra parida en posición inversa (flecha verde señala ovario con ovocitos, flecha roja traqueas, flecha azul traqueolas desenrolladas y flecha amarilla fragmento del tubo de Malpighi). B. Ovario izquierdo de hembra nulípara en posición inversa (flecha verde señala ovario con ovocitos, flecha roja tráqueas y flecha azul traqueolas enrolladas). C. Ovario izquierdo de hembra parida en posición opuesta a la inversa (flecha verde señala ovario con ovocitos, flecha roja tráqueas y flecha azul traqueolas desenrolladas). D. Ovario izquierdo de hembra nulípara en posición opuesta a la inversa (flecha verde señala ovario con ovocitos, flecha roja tráqueas y flecha azul traqueolas enrolladas). E. Ovario de hembra subgravida (flecha verde señala ovarios con ovocitos fecundados). F. Huevos desarrollados en hembra grávida. En A, B, C y D, los ovocitos no fecundados se diferencian de los fecundados por color translúcido y el tamaño.

### Identificación molecular de la resistencia a organofosforados y carbamatos

En la figura 2 se aprecia la amplificación del gen ACEr en el 100% (n=58) de los mosquitos hembras que se seleccionaron para el análisis molecular, indicando la resistencia a insecticidas organofosforados y carbamatos.

**Figura 2:** Electroforesis en gel de agarosa al 2% de los productos de la PCR dirigida al gen ACEr obtenido de especímenes adultos hembras de *Aedes aegypti* del cementerio San José de Sullana del norte de Perú. P1: Pool de 8 hembras grávidas. P2: Pool de 10 hembras grávidas. P3 y P4: Pool de 10 hembras paridas. P5 y P6: Pool de 10 hembras nulíparas.

## DISCUSIÓN

En el presente estudio se observó que más del 50% de las hembras analizadas presentaron traqueolas desenrolladas y algunas se hallaron grávidas. Las hembras clasificadas como nulíparas (juveniles) son aquellas sin alimentación con traqueolas enrolladas, mientras que las hembras paridas (longevas) aquellas que han completado una ovoposición y presentan traqueolas desenrolladas ^14^. La edad fisiológica de mosquitos culícidos puede estimarse mediante el análisis de la estructura traqueolar de sus ovarios ^15^. Según Cabezas ^10^, una hembra que se alimenta completamente de sangre pondrá hasta 200 huevos, en un intervalo de dos a tres días. Badil *et al*. ^16^ señalan que en *Aedes aegypti*, el intervalo entre la ingesta de sangre y la oviposición puede ser tan corto como tres días, bajo condiciones óptimas de temperatura y disponibilidad de huéspedes. Morin *et al*. ^17^, resaltan que la ingesta de sangre en los mosquitos influye directamente en la duración de los ciclos gonadotróficos, por lo que la distensión abdominal durante la alimentación es un factor clave. No obstante, estudios previos señalan que la digestión sanguínea está influenciada por la temperatura y la humedad, de modo que en temperaturas bajas el ciclo gonadotrófico se alarga, mientras que en temperaturas altas se acorta ^18^. Salas ^19^, describe qué, el ciclo gonadotrófico de *Aedes aegypti* puede durar hasta cinco días, aunque otros estudios sugieren que puede variar hasta 20 días ^20^.

En lo que corresponde a la detección del gen ACEr, este se encontró en 20 hembras nulíparas (juveniles), 20 paridas (longevas) y 18 grávidas de las paridas (longevas). Este hallazgo concuerda con resultados de Loaiza ^9^, quien detectó el mismo gen en poblaciones de *Aedes aegypti* en México. Un estudio similar demostró que poblaciones de *Aedes aegypti* presentan resistencia a insecticidas como deltametrina, permetrina, malatión, clorpirifos, temefos y bendiocarb, atribuida a una elevada actividad de enzimas detoxificantes, como el citocromo P450 y el glutatión S-transferasa, así como a una disminución en la sensibilidad de la enzima acetilcolinesterasa ^21^. De acuerdo con Sánchez y Salceda ^22^, la acetilcolinesterasa (AChE) es una enzima clave para el funcionamiento del sistema nervioso en insectos y otros organismos, su principal función es descomponer la acetilcolina, un neurotransmisor esencial para la transmisión de impulsos nerviosos en las sinapsis. Según Pascual y López ^23^, en los insectos, la AChE se localiza en las neuronas y en las placas motoras, donde regula la señalización neuronal.

En 2024, en Colombia, se evaluó la susceptibilidad a organofosforados y los mecanismos de resistencia en poblaciones de *Aedes aegypti*, hallando una resistencia a los insecticidas temefos, malatión, pirimifos metil y fenitrotión inducida por una insensibilidad causada por mutaciones en genes codificantes de las enzimas glutatión S-transferasas, α-esterasas, oxidasas de función mixta y acetilcolinesterasa ^24^. En 2021, en Panamá, se identificaron poblaciones de *Aedes aegypti* y *Aedes albopictus* que presentan resistencia a insecticidas de las clases piretroides, carbamatos y organofosforados ^25^. En Venezuela, se ha reportado la alteración en la expresión de enzimas implicadas en la actividad funcional de los insecticidas, como la glutatión-S-transferasa y la acetilcolinesterasa, lo que ha conducido al desarrollo de resistencia a organofosforados en *Anopheles aquasalis*, el principal vector de la malaria en ese país ^26^. Un estudio realizado en Cuba en 1986 reportó un aumento en las densidades de *Culex quinquefasciatus* en áreas urbanas, lo que posteriormente llevó a la identificación de resistencia al malatión en dos poblaciones de este mosquito ^27^. Por tanto, los resultados de este estudio, junto con informes de otros países sudamericanos y con el reporte de 2024 del Instituto Nacional de Salud (INS) del Perú sobre la resistencia de *Aedes aegypti* al malatión ^8^, refuerzan y complementan los hallazgos previamente descritos por el INS.

La fortaleza del estudio se sustenta en que, al usar técnicas moleculares en estrategias del control vectorial de *Aedes aegypti*, será posible realizar un monitoreo genético para detectar la aparición de mutaciones en los genes relacionados con la resistencia a insecticidas, logrando anticipar la necesidad de reemplazarlos y/o rotarlos por insecticidas con mayor eficacia.

La presente investigación tiene una limitación importante que debe reconocerse. Las capturas entomológicas se llevaron a cabo en un cementerio ubicado en una zona urbana, lo cual restringe la posibilidad de generalizar que las poblaciones de *Aedes aegypti* de cementerios de áreas rurales también presenten características genéticas asociadas a la resistencia.

En conclusión, en el norte de Perú, específicamente en el cementerio San José de Sullana del departamento de Piura, se ha identificado la existencia de poblaciones de *Aedes aegypti*, tanto adultas juveniles (nulíparas) como longevas (paridas y grávidas), que poseen una mutación en la acetilcolinesterasa, lo que les confiere resistencia a insecticidas organofosforados y carbamatos.
